# Nutritional, sensory, physico-chemical, phytochemical, microbiological and shelf-life studies of natural fruit juice formulated from orange (*Citrus sinensis*), lemon (*Citrus limon)*, Honey and Ginger (*Zingiber officinale*)

**DOI:** 10.1016/j.heliyon.2021.e07177

**Published:** 2021-05-29

**Authors:** Bernard Tiencheu, Desdemona Njabi Nji, Aduni Ufuan Achidi, Agbor Claudia Egbe, Noel Tenyang, Eurydice Flore Tiepma Ngongang, Fabrice Tonfack Djikeng, Bertrand Tatsinkou Fossi

**Affiliations:** a Department of Biochemistry and Molecular Biology, Faculty of Science, University of Buea, P.O. Box 63, Buea, Cameroon; b Department of Biological Science, Faculty of Science, University of Maroua, P.O. Box 814, Maroua, Cameroon; c School of Agriculture and Natural Resources, Catholic University Institute of Buea, P.O BOX 563, Buea, Cameroon; d Department of Microbiology and Parasitology, Faculty of Science, University of Buea, P. O. Box 63, Buea, Cameroon

**Keywords:** *Citrus sinensis*, *Citrus limon*, *Zingieber officinale*, Honey, Phytochemicals, Fruit drink, Shelf-life, Nutritional, Microbiological

## Abstract

Orange (*Citrus sinensis*), lemon (*Citrus limon)*, ginger (*Zingiber officinale*) and honey contain nutrients and phytochemicals that are beneficial to health. Most of the available fruit drinks are artificial and may contain a lot of chemicals which could be unhealthy and detrimental to the health of the consumers. This work was aimed at formulating a healthy fruit drink from the combination (blend) of orange, lemon, ginger and honey for the development of a new product. Thirty (30) different juice blends (formulae) were made and subjected to sensory evaluation, from which five best formulated juices were selected using a 9 point hedonic scale. These were then subjected to physicochemical, nutritional, phytochemical, microbiological analyses and shelf-life studies. The results of the study showed pH (3.40–4.90), Vitamin C (0.04–0.06 mg/mL), titrable acidity (0.04–0.21 citric acid mg/100mL), total soluble solids (2.90–20.69%), reducing (0.41–1.44 mg/mL) and non-reducing sugar (0.21–2.06 mg/mL). The moisture, protein, fat, ash, fibre and available carbohydrate contents ranged from 79.31-97.10 %, 0.01–0.56g/100mL, 0.05–0.11g/100mL, 0.51–1.13g/100mL, 0.01–0.09g/100mL and 16.39–22.99g/100mL respectively. The macro (K, Ca, P, Na) and micro (Zn, Fe) minerals varied differently with Potassium (K) being the most abundant. Amongst the five best formulated juices, F22 (5% lemon juice) was the most organoleptically accepted. On the other hand, F21 (10% lemon juice + sugar) which had the least overall acceptability amongst the five, was shown to be the most nutritive.

## 1 Introduction

Fruits and their juices are among of the most important foods for human, as their consumption maintains good health and replaces the losses in nutrients by the body (Ohwesiri et al., 2016). Fruit juice is nutritious and plays a crucial role in a healthy diet because it offers a variety of micronutrients found in earth (Nelofer et al., 2015). In rural areas of Cameroon, fresh fruits are consumed within short periods of seasonal availability after picked from wild trees or after harvesting. These fruit are carrying mainly from rural to urban areas or are mostly imported from neighbouring countries periods of abundance. The purchasing rate or power of consumers thus depends to some extent on their socio-economic status (Gouado et al., 2005). Citrus fruits (lemon and orange) are the most important tropical fruits widely grown all over the world and the second largest by production volume next to banana (FAO, 2004). In Cameroon, these fruits are consumed in several forms: fresh sliced (Gouado et al., 2005), added to dishes and beverages or processed into natural juice (Manner et al., 2006).

Orange, ginger, lemon and honey have been used as natural flavouring agent, preservative, stabilizer during several food formulations and confectionaries because of their aroma, sugar or acidity (Manner et al., 2006). A combination of these ingredients will yield a highly nutritive beverage or juice of unique flavour. Blended fruit juice can be formulated from several fruits such as lemon, orange and pineapple among others in order to diversify nutrients and avoid their deficiencies or completed absence. This will give a better quality juice with regards to the nutritional and organoleptic properties (Vwioko et al., 2013). Moreover, this could lead to a development of a new natural drink with health benefits (Ohwesiri et al., 2016).

A good number of natural as well as synthetic preservatives can be used to protect food from deterioration. Benzoic acid, citric acid, acetic acid, together with carbon dioxide and sulphite are acid preservatives used in food and soft drink preservation (Glevitzky et al., 2009). The condition created by these preservatives (low pH and lack of oxygen) are inconvenient for the growth of most microorganisms. In most cases, some resistant microbes such as yeast and other fungi may still grow even when good hygienic conditions are kept during food processing (Glevitzky et al., 2009).

Food preservative is a substance that inhibits, delay or stops the growth of microbe or any deteriorative compound due to the action of microorganism (Delores et al., 2004). The aim of preserving food is to prolong its shelf life. Some methods used in the shelf life extension process include water removal, freezing, drying, pH control, irradiation, vacuum packaging, acidification, pasteurization and addition of synthetic preservative. These processes are aimed at slowing down the changes that cause food spoilage due to a large number of biological, physical, enzymatic and chemical reactions (Mccoy, 2011).

Ginger has been extensively used as a natural preservative (Gundogdu et al., 2009). Citric acid, a natural preservative, exists naturally in citrus fruits, especially in lemon (FAO, 2004). Undiluted honey with its high sugar content is also a good natural preservative (Krushna et al., 2005). Natural preservatives are safer, and are good alternatives to chemical preservatives, thus minimizing the possible side effects associated with synthetic chemical preservatives (Mishra and Behal, 2010). Extract from ginger has been proved to prolong the shelf-life of *zobo* liquor (“fulere” drink) for up to two weeks while lime has been shown to be active against a litany of bacteria present in *zobo* liquor. In addition to these natural preservatives, chemical preservatives may be added to ensure maximum preservation for a longer period of time (Vwioko et al., 2013).

Orange, lemon, ginger and honey are rich in citric acid and have been used as natural preservatives in fruit drinks and other fruit products. Despite the acidity of citrus fruits like lemon and orange they are still destroyed by yeast and mould. Some examples of yeast resistant microbes include; *Aspergillus, Penicillium* and *Saccharomyces* (Glevitzky et al., 2009). However, quality fruit juice needs to be safe for consumption for as long as 3–9 months (Talasila et al., 2012). This can be realised by the combined effect of both natural and synthetic preservatives (SCCP, 2005). This work therefore aims at studying the nutritional, organoleptic, physico-chemical, phytochemical, microbiological and shelf-life of natural fruit juice formulated from orange (*Citrus sinensis*), Lemon (*Citrus limon*), Honey and Ginger (*Zingiber officinale*).

## 2 Materials and methods

### 2.1 Sample collection

Healthy, mature, ripe lemon and orange fruits and fresh ginger were purchased from the Buea Central Market. Mouldy and wounded fruits were exempted to avoid contamination, changes in colour, taste or flavour of the juice. Honey (Banyo Natural honey) and other ingredients were purchased from shops in Molyko, Buea. The samples were transported in a polythene bag to the University of Buea Life Science Laboratory for juice formulation, sensory, physico-chemical, nutritional, microbiological and phyto-chemical analyses and shelf-life studies.

### 2.2 Sample preparation, juice extraction and formulation

#### 2.2.1 Sample preparation

The fruits (orange and lemon) were washed with clean tap water and rinsed twice in chlorinated water, then rinsed with distilled water. They were then weighed on an analogue weighing balance (OHAUS, CS200), peeled and reweighed. The lemon and orange juices were extracted separately using an electronic juice extractor (Citrus juicer, CJ625). Both juices were filtered with the use of a tea sieve; first the orange juice, then the lemon juice. They were then bottled and kept for formulation and pasteurisation.

The ginger on the other hand, was washed with clean tap water and also rinsed in chlorinated water. The rhizomes of the ginger were removed to ensure complete removal of soil. The ginger was weighed, chopped into smaller sizes and blended with 600mL of water (Supermont ®) using an electric blender (PHILIPS). The ginger due to its rich fibre content had much chaff hence, was squeezed and filtered, first with a 0.2μm sieve, before sieving with the tea sieve. This was also ready for formulation and pasteurisation.

Egg yellow colour (0.2g) (E102, E110) was dissolved in 2L of Supermont water. Five drops of green concentrated food colour (E102 (1.8%), E110 (0.4%)) was added to give an intermediate yellow colour close to the colour of natural lemon and orange juices. The coloured water was used to complete each formulation to 1500mL (1.5L). In all, 0.5g of the egg yellow (E102, E110) colour was dissolved in 5L of water to which 12 drops of green concentrated food colour was added.

#### 2.2.2 Juice formulation

Thirty (30) juice formulations (recipes) (Table 1) were made ranging from 5 to 50% orange or lemon fruit base and were labelled from F1 to F30 with F1 (Brand A®) and F2 (Brand B®) being the commercials Brands or control juices purchased from the shop and F29 the locally homemade called “Brand C” of equal volume (25%/25%) orange lemon and sugar. These formulations were done thrice (3 batches) using a formulation table (Table 1) but mixed to obtain a single formula for the continuation. The juice was pasteurized at 90 °C for 10 min. It was allowed to cool for 45 min and filled into sterile labelled bottles. This was used for sensory, physico-chemical, nutritional, phytochemical, microbial analyses and shelf-life studies.

**Table 1:** Formulation table before sensory evaluation.

Juice Code	Orange	Lemon	Ginger	Honey	Table sugar	Vanilla sugar	Brand A	Brand B	Brand C	Water	Total (mL)
F1							100				100
F2								100			100
F3	50		5	10	5					30	100
F4	40		5	10	5					40	100
F5	30		5	10	5					50	100
F6	20		5	10	5					60	100
F7	10		5	10	5					70	100
F8	5		5	10	5					75	100
F9	50		5	10		5				30	100
F10	40		5	10		5				40	100
F11	30		5	10		5				50	100
F12	20		5	10		5				60	100
F13	10		5	10		5				70	100
F14	5		5	10		5				75	100
F15	25		5	10	5					55	100
F16		25	5	10		5				55	100
F17		50	5	10	5					30	100
F18		40	5	10	5					40	100
F19		30	5	10	5					50	100
F20		20	5	10	5					60	100
F21		10	5	10	5					70	100
F22		5	5	10	5					75	100
F23		50	5	10		5				30	100
F24		40	5	10		5				40	100
F25		30	5	10		5				50	100
F26		20	5	10		5				60	100
F27		10	5	10		5				70	100
F28		5	5	10		5				75	100
F29									100		100
F30		25	5	10						60	100

### 2.3 Sensory evaluation of formulae

Sensory evaluation tests were done on the thirty (30) formulated juices (included two standard commercial juices F1: Brand A and F2: Brand B used as control) to be able to choose those that were the best, based on consumers (panelists) acceptance test using a 9-point hedonic scale. For that purpose, colour, taste, flavour, texture and acceptability of the formulated juice was assessed by 30 semi-trained panellist members (Balaswamy et al., 2013), from whom informed consent was initially obtained. consisting of students, lecturers and staff around the university campus. The panellists were oriented prior to the sensory tests. The juices were coded and presented to the panellists individually, thereby preventing any interaction between panellists which could influence their responses. Each of the panellists was comfortably seated in an individual boot equipped with light and free from distractions. The panelists were recruited based on their acquaintance to juice consumption, and evaluated the samples for taste, colour, flavour, texture and overall acceptability using a nine point hedonic card, where 1 was the lowest score and 9 the highest. The degree to which a product was liked was scored as 9; 8; 7; 6; 5; 4; 3; 2 and 1 points, to express respectively: like extremely, like very much, like moderately, like slightly, neither like nor dislike, dislike slightly, dislike moderately, dislike very much and dislike extremely. The panellists were advised to take just 20ml of each of the coded samples. Each panellist was given a cup, a teaspoon and clean water to rinse their mouths in-between testing of each sample to avoid carry over effect. Ambient temperature of 25 °C was maintained throughout the testing duration. The best selected formulae among the 30 were subjected to the continuation of the studies (further analyses).

### 2.4 Analysis of best formulated juice selected after sensory evaluation

#### 2.4.1 Physico-chemical analysis

##### 2.4.1.1 Determination of pH

pH was determined in ten milliliters of the juice dispensed into a beaker after calibration with phosphate buffer of pH 4.0 and 7.0 (Adubofuor et al., 2010).

##### 2.4.1.2 Determination of total titratable acidity (TTA)

For the measurement of the titratable acidity the standard method of Talasila et al. (2012) was used. Five grams of concentrated fruit juice was diluted with distilled water (20ml) and filtered using filter paper (Whatman No. 1). The indicator (two drops of phenolphthalein) was added to 20ml of the filtrate and titrated against 0.05 M NaOH. The Total Titratable Acidity was calculated (Eq. (1)).

(1) TA = (M_NaOH_ x C_NaOH_ x 0.064 × 100)/V

Where: TA: Titratable acidity; M_NaOH_: Molarity of NaOH used; V_NaOH_ volume of NaOH used; 0.064: Equivalent weight of citric acid V: volume of juice.

##### 2.4.1.3 Determination of total solids (TSS)

Total solids content was determined by weighing an empty filter paper and then passing a known weight of juice through a Whatman No. 1 filter paper that retained particle or solids. After drying inside a ventilated oven at 103 °C for 2h, the solid left on the filter after evaporation was weighed and used to calculate the TSS (material remaining on the filter after moisture have been evaporated) (AOAC, 2005) as illustrated in Eq. (2).

(2) % Total solids = (W_2_ x 100)/W_1_= (100 - % moisture)

Where, W_1_: Initial weight; W_2_: Dried weight.

#### 2.4.2 Proximate, minerals and vitamin C analyses

##### 2.4.2.1 Proximate and minerals analysis

The method described by Association of Official Analytical Chemist (AOAC 2005) was used to evaluate the proximate analysis of the samples: Moisture content was determined gravimetrically, while crude fiber, ash, fat and protein, were determined according to the AOAC method (2005). Carbohydrate content was determined mathematically (Eq. (3)) by subtracting from 100, the percentage moisture, ash, protein, fat, and crude fiber. Na, P, Zn, Mg, Ca, Fe and K were analysed using the Association of Official Analytical method (2005).

(3) % carbohydrate = 100 – (% moisture + % ash + % protein + % fat + crude fiber).

##### 2.4.2.2 Determination of vitamin C

Vitamin C content was determined with the dichlorophenol-indophenol (DCP) method of Covenin (AOAC, 2005) with a slight modification.

###### 2.4.2.2.1 Standardization of 5 mL DCP with ascorbic acid

9.7 mg of pure vitamin C (C_6_H_8_O_6_) were accurately weighed out, dissolved with 50 mL of distilled water and stirred enough to dissolve all of the ascorbic acid. 5 mL of the DCP was accurately pipetted into a 50 mL Erlenmeyer flask, 1drop of acetic acid (30%) was added to change the blue colour of DCP to a pink colour. Ascorbic acid solution was used to titrate the DCP o a colourless endpoint (or equivalence point) using burette. The volume of ascorbic acid used was recorded and the titration repeated. The quantity of vitamin C that changed the color of DCP was then calculated.

###### 2.4.2.2.2 Titration of DCP with fruit juice

Standardization process was repeated by replacing ascorbic acid solution with 5ml of juice made up to 10 mL with distilled water. After repeating the titration 2 times, the vitamin C content was calculated from standard volume and expressed as mg ascorbic acid/100ml of juice.

#### 2.4.3 Phytochemical analysis

Both quantitative and qualitative analyses were carried out. The presence of major antioxidant secondary metabolite classes, namely, saponins, alkaloids, flavonoids, tannins, phenolics, and terpenoids were determined using standard phytochemical methods with some modifications (Iqbal et al., 2015).

##### 2.4.3.1 Quantitative evaluation of total phenolic content (TPC)

The TPC of the methanolic extracts was determined using the Folin- Ciocalteu colorimetric method described by Chlopicka et al. (2012). 0.2mL of the juice were mixed in a test tube with 1.5mL of Folin-Ciocalteu reagent. After incubated at room temperature for 5 min, 1.5mL of 6% sodium carbonate solution was added to the mixture and re-incubated at room temperature for 90 min. The absorbance of the resulting blue colour was measured using a quartz cuvet at 725 nm. Gallic acid standards were prepared as follow: In 5mL of distilled water, 0.75mg of gallic acid was dissolved. Into four labelled test tubes, different volumes (0, 0.1, 0.2, 0.3) of the gallic acid standard were pipetted. Water was added to complete the volumes to 0.3mL. To this, 2.25mL of Folin-Ciocalteu reagent was added and incubated at room temperature for 5 min. Finally, 2.25mL of 6% sodium carbonate solution was added and re-incubated for 90 min at room temperature. The absorbance of the resulting blue colour was read using a quartz cuvette at 725 nm and used to plot a standard curve from which the total phenolic content of the samples was estimated and expressed as milligram gallic acid equivalents (GAE) per 100mL of sample.

##### 2.4.3.2 Test for saponins (Frothing test)

Saponins were identified according to the method described by Banso and Adeyemo (2006). This was done by mixing 0.5mL of the juice in a test tube containing 3mL of hot distilled water, following by continuous vigorously shaking (1 min) to observe for persistent foaming.

##### 2.4.3.3 Test for flavonoids (Cyanidine test)

This was done according to the method of Stankovic (2011). Five hundred microliters (0.5mL) of juice was mixed with 2mL methanol and 1mL of concentrated sulphuric acid added. A spatula was used to add a powder of magnesium chloride (MgCl_2_) and the mixture observed for 1 min for effervescence and also observed for a brick red colouration.

##### 2.4.3.4 Test for steroids (Lieberman-Burchard test)

The method described by Joshi et al. (2013) was used. One millilitre (1mL) of each juice was dissolved in 2mL of chloroform. Three drops of acetic anhydride were added to the test tube and boiled in a water bath for 10 min. It was rapidly cooled in running tap water. Two (2mL) of Concentrated H_2_SO_4_ was added alongside. It was allowed to stand for 5 min for the development of a greenish colouration.

##### 2.4.3.5 Test for tannins (Ferric chloride test)

This was done according to the method of Banso and Adeyemo (2006). 500 microliters (0.5mL) of juice was added to a test tube containing 20mL of boiled distilled water and then heated for an hour. Five drops of ferric chloride were added and the tube was allowed to stand for colour development. A blue-black colouration indicated the presence of tannins.

##### 2.4.3.6 Test for alkaloids (Wagner's test)

This was done according to the method of Joshi et al. (2013). One millilitre (1mL) of juice was stirred with 0.4mL of 1% HCl in a water bath for 5 min and filtered. Two grams (2g) of Potassium iodide and 1.27g of iodine were dissolved in 5mL of distilled water and the solution was diluted to 100 mL with distilled water. Two drops of this iodine solution were added to the filtrate; a brown coloured precipitate indicated the presence of alkaloids.

##### 2.4.3.7 Test for cardiac glycosides (Keller-Killiani test)

It was done according to the method described by Ayoola et al. (2008). 500 microliters (0.5mL) of juice was added to 2 mL of glacial acetic acid containing two drop of ferric chloride. The set up was underplayed with 1 mL of concentrated sulphuric acid. It was observed for the appearance of violet and brownish rings below the interface, followed by the formation of a greenish ring in the acetic acid layer.

##### 2.4.3.8 Test for phenol

To 1 mL of each sample, one drop of 5% FeCl_3_ (w/v) was added. This was observed for 10 min for the formation of a greenish precipitate.

##### 2.4.3.9 Test for terpenoids (Salkowski test)

The method described by Ayoola et al. (2008) was used to test for terpenoids. To 0.2mL of chloroform, 0.5mL of juice was added. Concentrated H_2_SO_4_ (0.3mL) was carefully added to form a layer; the presence of terpenoids was indicated by the formation of reddish brown colouration at the level of the interface.

#### 2.4.4 Preservation and shelf life studies

Replicates of the best juices selected were treated with 25mg of sodium metabisulphite (preservative) in 50 mL of juice as recommended by Codex Alimentarius (2001) and coded by adding “P” on the original codes. The samples were kept at ambient temperature (25 ± 3 °C) and refrigeration conditions (4 °C) for 1 month. The physicochemical characteristics of the samples were evaluated at an interval of 7 days. The microbial load of the best preserved juice samples was determined. The total yeast, bacteria and coliform counts were carried out using Potato dextrose agar (PDA), Plate count agar (PCA) and Violet red bile lactose agar (VRBL) media respectively.

##### 2.4.4.1 Microbiological analyses

The total yeast, coliform and bacterial counts were carried out using the Potato dextrose agar (PDA), Violet red bile lactose agar (VRBL) and Plate count agar (PCA) media respectively (Olorunjuwon et al., 2014).

###### 2.4.4.1.1 Total yeast count

Potato Dextrose Agar (PDA) (4.2g) was dissolved in 100mL of distilled water. It was autoclaved at 121 °C for 1 h in an electric pressure steam sterilizer (Model No.25X) and allowed to cool to about 45 °C. The work environment was sterilised with 70% alcohol. 1mL of each sample was pipetted into labelled petri-dishes with the use of a micro pipette (Gilson Pipetman, 060087N). The medium (PDA) was poured into the Petri dish and agitated gently to homogenize with the sample. This solidified and formed a gel in the Petri dish. It was then incubated (DHP-9050) at 37 °C for 24 h. After 24 h, the total yeasts were counted as colony forming units (CFU).

###### 2.4.4.1.2 Total bacteria count

2.4g of Plate Count Agar (PCA) was dissolved in 100mL of distilled water. It was autoclaved at 121 °C for 1 h in an electric pressure steam sterilizer (Model No.25X) and allowed to cool to about 45 °C. The work environment was sterilised with 70% alcohol. 1mL of each sample was pipetted into labelled petri-dishes with the use of a micro pipette (Gilson Pipetman, 060087N). The medium (PCA) was poured into the Petri dish and agitated gently to homogenize with the sample. This solidified and formed a gel in the Petri dish. It was then incubated (DHP-9050) at 37 °C for 24 h. After 24 h, the total bacteria were counted as colony forming units (CFU).

###### 2.4.4.1.3 Total coliform count

Violet Red Bile Lactose Agar (VBRL) (6.2g) was dissolved in 150 mL of distilled water. This solution was allowed to boil while shaking over a Bunsen burner flame until it was completely dissolved. It was then allowed to cool to about 45 °C. The work environment was sterilised with 70% alcohol. One millilitre (1mL) of each sample was pipetted into labelled petri-dishes with the use of a micro pipette (Gilson Pipetman, 060087N). The medium (VBRL) was poured into the Petri dish and agitated gently to homogenize with the sample and allowed to set. More of the medium was poured to prevent entry of Oxygen. The solidified gel was then incubated (DHP-9050) at 42 °C for 24 h. After 24 h, the total coliforms were counted as colony forming units (CFU).

### 2.5 Statistical analysis

Raw data was computed using Microsoft EXCEL 2007. All data were presented as mean ± SD and was analysed using one-way analysis of variance (ANOVA) using Graphpad software to test the level of significance at 5% probability (p < 0.05). Bonferroni Test was used to separate the means where significant differences existed.

## 3 Results

### 3.1 Sensory (organoleptic) analysis of juice

The data for sensory analysis of the best formulated juice is shown on Table 2. Overall, the commercially packaged juice (F1: Brand A) and (F2: Brand B) scored highest with an overall acceptability of 6.70 and 6.53 respectively. This was closely followed by F22 (5% lemon juice) with an overall acceptability of 5.92. Amongst the five best samples (F8, F13, F21, F22 and F27), F21 (10% lemon juice) had the least overall acceptability.

**Table 2:** Results of organoleptic analysis.

Code	Juice Composition	Overall acceptability
F1	Brand A	6.70_a_ ± 0.00
F2	Brand B	6.53_a_ ± 0.04
F22	5% (v/v) lemon juice	5.92_a_ ± 0.02
F13	10% (v/v) orange juice	5.68_a_ ± 0.38
F8	5% (v/v) orange juice	5.63_a_ ± 0.32
F27	10% (v/v) lemon juice	5.57_a_ ± 0.16
F21	10% lemon juice (+sugar)	5.50_a_ ± 0.14

Results are expressed as mean ± standard deviation; a,b,c. Means with the different letter in the same column are significantly different at p < 0.05.

### 3.2 Physico-chemical analysis

The results of the physico-chemical analysis of the best formulated juices are presented on Table 3. The pH ranged from 3.40 in F21 to 4.90 in F8. The pH value for the orange (F8, F13) based samples were higher than the lemon-based samples (F21, F22, F27) with an indication that lemon is more acidic than orange. The vitamin C content ranged from 0.08-0.12 mg/mL. Though the orange-based samples F8 and F13 have higher pH values, they contain more Vitamin C than the lemon-based F21, F22 and F27. The titrable acidity ranged from 0.04-0.11 citric acid mg/100mL and the total soluble solids ranged from 1.68-3.16%. Generally, the lemon based samples (F21, F22 and F27) had a higher titrable acidity than the orange-based F8 and F13. The soluble solid content was higher in the orange based F8 and F13 than in lemon-based samples (F21, F22 and F27). The reducing and non-reducing sugar content ranged from 2.05 mg/mL in F27 (10% lemon juice) to 2.41 mg/mL in F13 (10% orange juice) and 1.35 mg/mL in F27 (10% lemon juice) to 2.25 mg/mL in F8 (5% orange juice) respectively. There was no significant difference (P ≥ 0.05) in the vitamin C and reducing sugar content of the juice. Whereas, a significant difference was observed in the titrable acidity, total soluble solids and non-reducing sugar content of the juice (P ≤ 0.05).

**Table 3:** Physico-chemical analysis of Juice.

Sample code	pH	Vitamin C content (mg/mL)	Titrable acidity (citric acid mg/100mL)	Total soluble solids (%)	Reducing sugar (mg/mL)	Non-reducing sugar (mg/mL)
F8	4.90	0.12_a_ ± 0.01	0.04_a_ ± 0.01	3.16_b_ ± 0.01	2.26_a_ ± 0.12	2.25_b_ ± 0.03
F13	4.70	0.11_a_ ± 0.01	0.04_a_ ± 0.01	2.90_b_ ± 0.01	2.41_a_ ± 0.15	2.06_b_ ± 0.15
F21	3.40	0.09_a_ ± 0.00	0.06_a_ ± 0.01	2.61_b_ ± 0.00	2.11_a_ ± 0.02	1.98_b_ ± 0.10
F22	3.60	0.08_a_ ± 0.01	0.08_b_ ± 0.00	1.85_a_ ± 0.00	2.14_a_ ± 0.04	1.79_b_ ± 0.02
F27	3.50	0.10_a_ ± 0.00	0.11_b_ ± 0.04	1.68_a_ ± 0.00	2.05_a_ ± 0.01	1.35_a_ ± 0.01

Results are expressed as mean ± standard deviation; a,b, Means with the different subscript letter in the same column are significantly different at p < 0.05.

### 3.3 Proximate composition of juice

The proximate composition of the best formulated juices is shown on Table 4. The moisture content of the juices ranged from 79.31% in F27 to 97.10% in F13. The protein content ranged from 0.01 g/100mL in F13 and F22 to 0.56 g/100mL F8. The carbohydrate content ranged from 16.39 g/100mL in F27 to 22.99 g/100mL in F21. The fat content ranged from 0.05 g/100mL in F22 to 0.11 g/100mL in F8. Also, there was a significant difference between treatment means at 95% confidence interval. The fibre content ranged from 0.01 g/100mL in F8 to 0.09 g/100mL in F21. Except for F22, the lemon-based samples (F21, F27) contained more fibre than the orange based F8 and F13. Meanwhile, the mineral (ash) content ranged from 0.51 g/100mL in F13 and F21 to 1.13 g/100mL in F22 with no significant difference at 95% confidence interval (P ≥ 0.05) for both fibre and ash content. Generally, the samples were rich in carbohydrate and moisture, but low in protein, fibre, fat and ash.

**Table 4:** Proximate composition of juice.

Code	Moisture content (%)	Protein (g/100mL)	Carbohydrate (g/100mL)	Fiber (g/100mL)	Fat (g/100mL)	Ash (g/100mL)
F8	96.84_a_ ± 0.00	0.56_b_ ± 0.00	21.83_a_ ± 0.48	0.01_a_ ± 0.00	0.11_a_ ± 0.01	0.63_a_ ± 0.00
F13	97.10_a_ ± 0.36	0.01_a_ ± 0.00	20.30_a_ ± 0.99	0.02_a_ ± 0.01	0.10_a_ ± 0.00	0.51_a_ ± 0.00
F21	96.84_a_ ± 0.00	0.02_a_ ± 0.00	22.99_a_ ± 1.34	0.09_a_ ± 0.00	0.10_a_ ± 0.01	0.51_a_ ± 0.01
F22	82.45_b_ ± 1.86	0.01_a_ ± 0.00	20.15_a_ ± 2.04	0.02_a_ ± 0.00	0.05_b_ ± 0.01	1.13_b_ ± 0.01
F27	79.31_b_ ± 2.58	0.06_a_ ± 0.00	16.39_b_ ± 1.99	0.07_a_ ± 0.00	0.10_a_ ± 0.01	1.02_b_ ± 0.00

Results are expressed as mean ± standard deviation; a,b, Means with the different letters in the same column are significantly different at p < 0.05.

### 3.4 Mineral analysis of juice

Mineral content of the best formulated juice is presented on Table 5. The table revealed that Potassium and Calcium were mainly represented in all the formulated juices; while Zinc exhibited the lowest amount. In addition to those two minerals, F8 also have an appreciable quantity of Phosphorus and Magnesium, F13 in Iron, Zinc and Sodium while F22 was generally low in other minerals. There was a significant difference between the means at 95% confidence interval (P ≤ 0.05) since the formulated juices have different proportions in term of ingredients.

**Table 5:** Mineral composition of juice.

Mineral (mg/mL)	F8	F13	F21	F22	F27
P	0.12_c_ ± 0.02	0.08_b_ ± 0.04	0.10_b_ ± 0.01	0.01_a_ ± 0.00	0.06_b_ ± 0.00
Fe	0.06_b_ ± 0.01	0.10_b_ ± 0.03	0.07_b_ ± 0.01	0.06_b_ ± 0.02	0.06_b_ ± 0.01
Ca	0.21_c_ ± 0.05	0.19_c_ ± 0.00	0.20_c_ ± 0.03	0.19_c_ ± 0.00	0.24_c_ ± 0.00
Mg	0.07_b_ ± 0.01	0.06_b_ ± 0.03	0.06_b_ ± 0.02	0.05_b_ ± 0.00	0.04_a_ ± 0.01
Zn	0.01_a_ ± 0.00	0.02_a_ ± 0.01	0.01_a_ ± 0.00	0.01_a_ ± 0.00	0.01_a_ ± 0.00
K	0.23_c_ ± 0.08	0.23_c_ ± 0.08	0.29_c_ ± 0.00	0.23_c_ ± 0.08	0.23_c_ ± 0.08
Na	0.08_b_ ± 0.03	0.10_b_ ± 0.00	0.08_b_ ± 0.03	0.06_b_ ± 0.00	0.06_b_ ± 0.00

Results are expressed as mean ± standard deviation; a,b,c, Means with the same letters in the same row are not significantly different at p > 0.05.

### 3.5 Phytochemical analysis

The qualitative and quantitative phytochemical parameters of the best formulated juice samples are presented on Tables 6 and 7. Qualitative phytochemical analysis (Table 6), revealed the presence of alkaloids, flavonoids, phenolics, saponins, terpenoids and tannins in all five juices with the absence of cardiac glycosides and steroids. The total polyphenol content ranged from 0.12 to 0.48mg GAE/mL (Table 7).

**Table 6:** Qualitative phytochemical analysis.

Code	Alkaloid	Cardiac glycosides	Flavonoid	Phenolic	Saponin	Steroid	Tannin	Terpenoid
F8	+	--	+	+	+	--	+	+
F13	+	--	+	+	+	--	+	+
F21	+	--	+	+	+	--	+	+
F22	+	--	+	+	+	--	+	+
F27	+	--	+	+	+	--	+	+

Present = +; absent = --

**Table 7:** Total polyphenol content.

Sample code	Total polyphenol count (TPC) (mg GAE/mL)
F8	0.12_a_ ± 0.02
F13	0.48_b_ ± 0.05
F21	0.40_b_ ± 0.03
F22	0.32_b_ ± 0.02
F27	0.31_b_ ± 0.05

Results are expressed as mean ± standard deviation; a,b, Means with the same letter in the same column are not significantly different at p > 0.05.

### 3.6 Preservation and shelf-life studies

#### 3.6.1 Physicochemical analysis

The results for the physicochemical analysis of the best formulated juice after four weeks of storage with and without preservative both at room (25 ± 3 °C) and refrigeration (4 °C) temperatures are presented on Tables 8, 9, 10, 11, 12, and 13. A general decrease in pH, non-reducing sugar and vitamin C were observed with increase in storage time at both conditions. However, an increase in titrable acidity, soluble solids and reducing sugar was observed with increase in storage time.

**Table 8:** pH of juice after four weeks of storage.

Code	pH at room temperature (25 °C)				pH at refrigeration temperature (4 °C)	
	Week 1	Week 2	Week 3	Week 4	Week 2	Week 4
F8	4.50	4.20	4.00	X	4.30	4.00
F13	4.30	4.00	3.60	X	4.10	3.90
F21	3.30	3.20	3.10	X	3.40	X
F22	3.40	3.30	3.20	3.00	3.20	3.00
F27	3.30	3.20	3.20	X	3.20	3.00
F8p	4.30	4.00	3.90	X	4.30	4.10
F13p	4.20	4.00	4.00	3.40	4.20	3.90
F21p	3.60	3.40	3.40	3.30	3.40	3.30
F22p	3.50	3.30	3.20	3.00	3.20	3.00
F27p	3.40	3.20	3.10	X	3.20	3.00

P stands for formula with preservative; X: stands for samples that were already bad with mould development and were therefore not analysed or discarded.

**Table 9:** Vitamin C content of juice after four weeks of storage.

Code	Vitamin C content at room temperature (mg/mL)				Vitamin C content at refrigeration temperature (mg/mL)	
	Week 1	Week 2	Week 3	Week 4	Week 2	Week 4
F8	0.09_a_ ± 0.01	0.07_a_ ± 0.00	0.04_b_ ± 0.00	X	0.12_a_ ± 0.01	0.06_b_ ± 0.00
F13	0.08_a_ ± 0.01	0.06_a_ ± 0.01	0.03_b_ ± 0.00	X	0.11_a_ ± 0.02	0.04_b_ ± 0.00
F21	0.07_a_ ± 0.01	0.05_b_ ± 0.00	0.02_c_ ± 0.00	X	0.07_b_ ± 0.01	X
F22	0.05_b_ ± 0.00	0.04_b_ ± 0.00	0.02_c_ ± 0.00	0.01_c_ ± 0.00	0.08_b_ ± 0.00	0.03_a_ ± 0.00
F27	0.07_a_ ± 0.01	0.06_a_ ± 0.00	0.01_c_ ± 0.00	X	0.09_a_ ± 0.00	0.05_b_ ± 0.00
F8p	0.08_a_ ± 0.00	0.06_a_ ± 0.01	0.02_c_ ± 0.00	X	0.11_a_ ± 0.01	0.05_b_ ± 0.00
F13p	0.07_a_ ± 0.01	0.05_b_ ± 0.00	0.03_b_ ± 0.00	0.01_c_ ± 0.00	0.10_a_ ± 0.01	0.04_b_ ± 0.00
F21p	0.05_b_ ± 0.01	0.04_b_ ± 0.01	0.02_c_ ± 0.00	0.01_c_ ± 0.00	0.05_c_ ± 0.00	0.02_a_ ± 0.00
F22p	0.04_b_ ± 0.00	0.03_b_ ± 0.00	0.01_c_ ± 0.00	0.01_c_ ± 0.00	0.07_b_ ± 0.00	0.03_a_ ± 0.00

a,bc, Means with the same letter in the same column are not significantly different at p > 0.05.

P stands for formula with preservative; X: stands for samples that were already bad with mould development and were therefore not analysed or discarded.

**Table 10:** Titrable acidity of juice after four weeks of storage.

Code	Titrable acidity at room temperature (citric acid mg/100mL)				Titrable acidity at refrigeration temperature (citric acid mg/100mL)	
	Week 1	Week 2	Week 3	Week 4	Week 2	Week 4
F8	0.06_a_ ± 0.01	0.07_a_ ± 0.01	0.09_a_ ± 0.02	X	0.05_a_ ± 0.01	0.10_c_ ± 0.01
F13	0.05_a_ ± 0.01	0.06_a_ ± 0.01	0.07_a_ ± 0.01	X	0.05_a_ ± 0.01	0.06_a_ ± 0.02
F21	0.08_b_ ± 0.01	0.10_b_ ± 0.01	0.11_b_ ± 0.01	X	0.07_b_ ± 0.01	X
F22	0.10_c_ ± 0.01	0.12_b_ ± 0.02	0.15_c_ ± 0.02	0.17_d_ ± 0.02	0.09_b_ ± 0.01	0.11_c_ ± 0.02
F27	0.13_c_ ± 0.01	0.15_c_ ± 0.01	0.17_d_ ± 0.01	X	0.13_c_ ± 0.02	0.15_b_ ± 0.01
F8p	0.05_a_ ± 0.02	0.06_a_ ± 0.01	0.07_a_ ± 0.01	X	0.04_a_ ± 0.00	0.06_a_ ± 0.01
F13p	0.04_a_ ± 0.01	0.06_a_ ± 0.01	0.08_a_ ± 0.01	0.11_b_ ± 0.00	0.06_a_ ± 0.01	0.08_a_ ± 0.01
F21p	0.07_b_ ± 0.01	0.08_a_ ± 0.02	0.09_a_ ± 0.00	0.14_c_ ± 0.01	0.10_c_ ± 0.00	0.13_b_ ± 0.01
F22p	0.09_b_ ± 0.02	0.11_b_ ± 0.01	0.12_b_ ± 0.02	0.15_c_ ± 0.01	0.08_b_ ± 0.01	0.11_c_ ± 0.02
F27p	0.12_c_ ± 0.01	0.13_c_ ± 0.01	0.15_c_ ± 0.02	X	0.09_b_ ± 0.01	0.12_c_ ± 0.00

Results are expressed as mean ± standard deviation; a,b,c Means with the same letter in the same column are not significantly different at p > 0.05.

P stands for formula with preservative; X: stands for samples that were already bad with mould development and were therefore not analysed or discarded.

**Table 11:** Soluble solid content of juice after four weeks of storage.

Code	Soluble solid content at room temperature (%)				Soluble solid content at refrigeration temperature (%)	
	Week 1	Week 2	Week 3	Week 4	Week 2	Week 4
F8	5.87_a_ ± 0.10	7.10_a_ ± 0.21	2.79_b_ ± 0.03	X	4.14_a_ ± 0.20	5.10_a_ ± 0.14
F13	3.78_a_ ± 0.07	5.80_a_ ± 0.12	1.09_c_ ± 0.01	X	3.78_a_ ± 0.05	4.63_a_ ± 0.08
F21	3.70_b_ ± 0.05	4.73_a_ ± 0.03	1.01_c_ ± 0.00	X	2.80_b_ ± 0.03	X
F22	3.52_b_ ± 0.05	4.54_a_ ± 0.02	3.33_b_ ± 0.02	1.19_c_ ± 0.03	2.09_b_ ± 0.03	2.99_b_ ± 0.03
F27	3.22_b_ ± 0.01	4.24_a_ ± 0.01	1.02_c_ ± 0.01	X	2.02_b_ ± 0.01	2.79_b_ ± 0.02
F8p	4.55_a_ ± 0.16	4.57_a_ ± 0.03	1.67_c_ ± 0.00	X	3.84_a_ ± 0.05	4.67_a_ ± 0.10
F13p	3.14_b_ ± 0.54	3.95_b_ ± 0.01	2.94_b_ ± 0.03	1.33_c_ ± 0.01	3.04_a_ ± 0.01	3.19_a_ ± 0.02
F21p	2.41_b_ ± 0.01	2.45_b_ ± 0.00	2.05_b_ ± 0.00	1.14_c_ ± 0.01	2.78_b_ ± 0.03	3.67_a_ ± 0.05
F22p	2.93_b_ ± 0.00	3.41_b_ ± 0.00	2.68_c_ ± 0.05	1.08_c_ ± 0.02	2.05_b_ ± 0.00	2.15_b_ ± 0.01
F27p	2.21_b_ ± 0.04	2.33_b_ ± 0.03	0.91_a_ ± 0.00	X	2.40_b_ ± 0.01	3.10_a_ ± 0.01

Results are expressed as mean ± standard deviation; a,b,c, Means with the same letter in the same column are not significantly different at p > 0.05.

P stands for formula with preservative; X: stands for samples that were already bad with mould development and were therefore not analysed or discarded.

**Table 12:** Reducing sugar content of juice after four weeks of storage.

Code	Reducing sugar at room temperature (mg/mL)				Reducing sugar at refrigeration temperature (mg/mL)	
	Week 1	Week 2	Week 3	Week 4	Week 2	Week 4
F8	2.86_a_ ± 0.06	4.69_b_ ± 0.50	2.11_a_ ± 0.00	X	2.71_a_ ± 0.09	2.92_a_ ± 0.05
F13	2.71_a_ ± 0.12	3.97_b_ ± 0.30	2.11_a_ ± 0.00	X	2.64_a_ ± 0.00	2.84_a_ ± 0.02
F21	2.52_a_ ± 0.10	3.59_b_ ± 0.50	1.96_a_ ± 0.01	X	2.19_a_ ± 0.02	X
F22	2.24_a_ ± 0.03	2.94_a_ ± 0.02	1.03_b_ ± 0.00	1.00_b_ ± 0.01	2.20_a_ ± 0.01	2.62_a_ ± 0.01
F27	2.34_a_ ± 0.05	2.86_a_ ± 0.11	1.24_b_ ± 0.01	X	2.14_a_ ± 0.01	2.17_a_ ± 0.03
F8p	2.76_a_ ± 0.56	3.96_b_ ± 0.14	1.93_a_ ± 0.04	X	2.57_a_ ± 0.02	2.99_a_ ± 0.11
F13p	2.61_a_ ± 0.05	3.74_b_ ± 0.10	2.15_a_ ± 0.01	1.98_a_ ± 0.04	2.39_a_ ± 0.01	2.72_a_ ± 0.05
F21p	2.54_a_ ± 0.08	3.31_b_ ± 0.04	2.05_a_ ± 0.10	1.76_a_ ± 0.02	2.22_a_ ± 0.03	2.68_a_ ± 0.01
F22p	2.17_a_ ± 0.01	2.75_a_ ± 0.02	1.71_b_ ± 0.01	1.66_b_ ± 0.01	2.14_a_ ± 0.00	2.55_a_ ± 0.03
F27p	2.33_a_ ± 0.02	2.63_a_ ± 0.01	1.08_b_ ± 0.00	X	2.12_a_ ± 0.01	2.38_a_ ± 0.01

a,bc, Means with the same letter in the same column are not significantly different at p > 0.05.

P stands for formula with preservative; X: stands for samples that were already bad with mould development and were therefore not analysed or discarded.

**Table 13:** Non-reducing sugar content of juice after four weeks of storage.

Code	Non-reducing sugar at room temperature (mg/mL)				Non-reducing sugar at refrigeration temperature (mg/mL)	
	Week 1	Week 2	Week 3	Week 4	Week 2	Week 4
F8	2.21_b_ ± 0.01	2.17_b_ ± 0.06	2.10_b_ ± 0.12	X	2.24_b_ ± 0.15	2.21_b_ ± 0.11
F13	2.03_b_ ± 0.10	2.10_b_ ± 0.08	1.93_b_ ± 0.03	X	2.04_b_ ± 0.07	2.00_b_ ± 0.03
F21	1.75_a_ ± 0.03	1.71_a_ ± 0.02	1.51_a_ ± 0.01	X	1.83_b_ ± 0.02	X
F22	1.61_a_ ± 0.01	1.58_a_ ± 0.01	1.46_a_ ± 0.01	1.32_a_ ± 0.03	1.65_a_ ± 0.00	1.61_a_ ± 0.03
F27	1.29_a_ ± 0.11	1.28_a_ ± 0.05	1.24_a_ ± 0.02	X	1.33_a_ ± 0.05	1.29_a_ ± 0.04
F8p	2.23_b_ ± 0.09	2.20_b_ ± 0.06	2.16_b_ ± 0.15	X	2.25_b_ ± 0.51	2.22_b_ ± 0.06
F13p	2.02_b_ ± 0.12	2.00_b_ ± 0.06	1.86_b_ ± 0.00	1.12_a_ ± 0.01	2.03_b_ ± 0.17	1.99_b_ ± 0.03
F21p	1.72_a_ ± 0.08	1.69_a_ ± 0.10	1.68_a_ ± 0.03	1.49_a_ ± 0.50	1.82_b_ ± 0.28	1.56_a_ ± 0.03
F22p	1.59_a_ ± 0.10	1.48_a_ ± 0.03	1.37_a_ ± 0.02	1.15_a_ ± 0.01	1.63_a_ ± 0.06	1.33_a_ ± 0.01
F27p	1.30_a_ ± 0.04	1.24_a_ ± 0.01	1.19_a_ ± 0.01	X	1.31_a_ ± 0.03	1.25_a_ ± 0.00

a,bc, Means with the same letter in the same column are not significantly different at p > 0.05.

P stands for formula with preservative; X: stands for samples that were already bad with mould development and were therefore not analysed or discarded.

##### 3.6.1.1 pH

The pH ranged from 3.00-4.50 (Table 8). The orange based F8 and F13 (with or without preservative) had higher pH values than the lemon based F21, F22 and F27. The pH values for the juice stored at refrigeration temperature were slightly higher than those at room temperature (25 ± 3 °C). But these differences were not statistically significant (P ≥ 0.05).

##### 3.6.1.2 Vitamin C

The Vitamin C content of the juice ranged from 0.01-0.12 mg/mL (Table 9). The juices stored in the fridge had higher values than those stored at room temperature (25 ± 3 °C), though there was a general decrease in vitamin C content with increase in storage time at both room and refrigeration temperatures. The juices without preservative also had higher vitamin C values than those with preservative. There was a significant difference between treatment means at 95% confidence interval (P ≤ 0.05).

##### 3.6.1.3 Titrable acidity

The titrable acidity (Table 10) of the juice increased with increase in storage time as it ranged from 0.04-0.17 citric acid mg/100mL. The juices at room temperature (25 ± 3 °C) had higher values than those at refrigeration temperature. The lemon based F21, F22 and F27 (with and without preservative) had higher values than the orange based F8 and F13. A significant difference was observed at 95% confidence interval (P ≤ 0.05).

##### 3.6.1.4 Total soluble solids

The total soluble solid content (Table 11) of the juice ranged from 0.91-7.10%. It was higher in the juices stored at room temperature (25 ± 3 °C). The total soluble solid increased within the first two weeks of storage at room temperature whereas it decreased in the third and fourth week. In the juices stored at refrigeration temperature, the soluble solid content increased till the fourth week of storage. It was also higher in the orange based F8 and F13 than in the lemon based juices (F21, F22, and F27). There was a significant difference (P ≤ 0.05) in the soluble solids in the different samples within the storage period at both conditions.

##### 3.6.1.5 Reducing sugar

The reducing sugar ranged from 1.00-4.69 mg/mL (Table 12). The reducing sugar increased within the first two weeks of storage at room temperature. Meanwhile, it decreased in last two weeks of storage. The juices stored at room temperature had higher values than those stored at refrigeration temperature with a significant difference at 95% confidence interval (P ≤ 0.05). The orange based juices (F8, F13), also had higher reducing sugar values than the lemon based F21, F22 and F27. In addition, there were differences in values in both preserved and unpreserved juices but these differences were not statistically significant (P ≥ 0.05).

##### 3.6.1.6 Non-reducing sugar

The non-reducing sugar content of the juice ranged from 1.12-2.25 mg/mL (Table 13). It decreased with increase in storage time and was higher in the juices stored at refrigeration temperature. The non-reducing sugar was also higher in the orange based juices (F8, F13) than in the lemon based F21, F22 and F27. There was a significant difference between treatment means at 95% confidence interval (P ≤ 0.05).

#### 3.6.2 Microbial analysis of juice

The microbial content of the best formulated juices is presented on Tables 14 and 15. The fresh juice sample analysed before storage had no microorganism. After 4 weeks of storage, the samples were shown to be low in coliform and yeast but high in total bacteria. The total bacteria count ranged from 0.00 × 10^˗1^ to 3.00 × 10^2^ CFU/mL, total coliform from 0.00–8.00 × 10^˗1^ CFU/mL and total yeast count from 0.00 × 10^˗1^ to 1.20 × 10^2^ CFU/mL. F22w4p (sample with preservative) had the least amount of microbes at room temperature (Table 14) whereas F22 (sample without preservative) had the most. In the refrigerator (Table 15), the samples with preservative generally had a smaller number of microbes than those without preservative. Thus F8w4p, F21w4p and F27w4p (samples with preservative) had no microbes after 4 weeks of storage in the refrigerator.

**Table 14:** Microbial load of juice after four weeks of preservation at room temperature.

Sample code	Total bacteria count (CFU/mL)	Total coliform count (CFU/mL)	Total yeast count (CFU/mL)
F13w4p	1.08 × 10^2^	0.00 × 10^˗1^	8.8 × 10
F21w4p	7.40 × 10	0.00 × 10^˗1^	7.50 × 10
F22	3.00 × 10^2^	0.00 × 10^˗1^	1.20 × 10^2^
F22w4p	7.50 × 10	0.00 × 10^˗1^	6.00 × 10

**Table 15:** Microbial load of juice after preservation at 4 °C.

Sample code	Total bacteria count (CFU/mL)	Total coliform count (CFU/mL)	Total yeast count (CFU/mL)
F8	5.50 × 10	8.00 × 10^˗1^	5.20 × 10
F13	3.80 × 10	0.00 × 10^˗1^	5.10 × 10
F22	7.00 × 10^˗1^	0.00 × 10^˗1^	0.00 × 10^˗1^
F27	1.70 × 10	0.00 × 10^˗1^	0.00 × 10^˗1^
F8w4p	0.00 × 10^˗1^	0.00 × 10^˗1^	0.00 × 10^˗1^
F13w4p	2.20 × 10	2.00 × 10^˗1^	0.00 × 10^˗1^
F21w4p	0.00 × 10^˗1^	0.00 × 10^˗1^	0.00 × 10^˗1^
F22w4p	6.00 × 10^˗1^	0.00 × 10^˗1^	0.00 × 10^˗1^
F27w4p	0.00 × 10^˗1^	0.00 × 10^˗1^	0.00 × 10^˗1^

P stands for formula with preservative.

## 4 Discussion

In this study, the sensory evaluation results (Table 2) of formulated juices from extracts from orange, lemon, ginger and honey in comparison with commercial juice revealed that commercially packaged juice had the highest overall acceptability. Also, they were most preferred for colour and taste. This could be due to the addition of artificial colour and sweeteners (Oluseyi, 2003). The colour of fruit when attractive during storage influenced the other sensory characteristics (Yau et al., 2010). However, the blending of the natural flavours of ginger, lemon, orange and honey generally gave a unique and better flavour than the commercially packaged juice. In addition, the rich phytochemical and nutrient content of the natural juice can make them better than the commercially packaged juice.

The pH of fruit juice is dependent on the maturity and stage of ripeness of the fruits used in the production (Olumuyiwa et al., 2003). The results of the physico-chemical analysis (Table 3) revealed a pH range of 3.40–4.90. This falls within the accepted range of 2–5 for fruit and vegetable juices (Tasnim et al., 2010). It is also, in line with reports from Adubofuor et al. (2010) (4.82–4.99) and Ndife et al. (2013), (3.23–4.08) for different brands of fruit juices. Fruit juices generally have low pH because they are comparatively rich in organic acid. More so, lemon and orange are rich in citric acid (Tasnim et al., 2010). The reverse was observed in the values of titrable acidity (Ndife et al., 2013; Ohwesiri et al., 2016). This indicates that, as pH value decreased, juices get more acidic. In citrus fruits, the dominant acid in lemon and orange juice is citric acid as reported by Kareem and Adebowale (2007). This citric acid is more abundant in lemon (F27:0.11mg/100mL; F22:0.08mg/100mL; F21:0.06mg/100mL) than in orange (F8, F13:0.04mg/100mL) juice and could give lemon its protective power against kidney stones (Nelofer et al., 2015). Though increase in titrable acidity reflects a decreased pH, the titrable acidity determines the acid taste in the juice whereas the pH determines its susceptibility to microbial spoilage (Tasnim et al., 2010).

Vitamin C plays an antioxidant role and possesses several health benefits (May and Qu, 2005). Ascorbic acid is used not only to fortify food and losses during processing, but also contributes to the product stability and appearance (Tasnim et al., 2010). This study revealed that, the vitamin C content ranged from 0.08-0.12 mg/mL (Table 3), which are fairly higher than those reported by Cook (2009) for water melon juice and can meet the Recommended Daily Intake as reported by (Adedeji et al., 2014). This is of great health importance and indicates that the juice can successfully been used in vitamin C deficiency and scurvy (Edem and Miranda, 2011). The consumption of vitamin C has also been reported to improve the rate of transformation of cholesterol, to prevent cancers and disorders associated with a lack of collagen (May and Qu, 2005).

The values for total solids obtained in this study are lower than results obtained by other researchers: 11.75–17.53% for 100% pineapple juice (Ohwesiri et al., 2016), 7.22–9.28% for cocktail juices (Adubofuor et al., 2010) and 5.50–11.80% for different brands of orange juice samples (Ndife et al., 2013). It was higher in the orange based juices than in the lemon in line with reports from Nelofer et al. (2015). The variations observed could be attributed to the blends of different fruit types (Ohwesiri et al., 2016) and to the difference in refractometer measurement as opposed to oven drying method used in this study. The Federal Institute of Industrial Research, Oshodi, (FIIRO) reported that the differences in production processes may explain most differences observed in juice composition and quality (FIIRO, 2005).

Generally, a high reducing and non-reducing sugar content were observed. This could be due to the presence of the sugar-rich honey in the juice formulation and the conversion of polysaccharides to monosaccharides and oligosaccharides (Ijah et al., 2015). This is fairly higher than reports obtained by Tasnim et al. (2010) for mango and orange juices of different companies.

The moisture content (Table 4) of the juices in this study is in close relationship with reports from Ohwesiri et al. (2016) for orange/pineapple juice blends (82.48–88.35%). According to Ponnusha et al. (2011), high moisture content promotes susceptibility to microbial activity though this is usually reduced with the use of preservatives. The protein content of the fruit juices was generally low. The general low protein content of fruit juices has also been reported for orange/pineapple juice blends and fresh beetroot juice (Ohwesiri et al., 2016; Emelike et al., 2015). According to Emelike et al. (2015) fruit juices are poor in proteins. The carbohydrate content (Table 4) is higher than a reported range of 8.16–16.19% (Ohwesiri et al., 2016) for orange/pineapple juice blends and 7.3% (Emelike et al., 2015) for fresh beetroot juice. This variations observed in these values may be associated with the difference in fruit types used. The ash (mineral) content (0.51–1.13%) of the juices was similar to the range of 0.42–2.68% for orange/pineapple juice blends (Ohwesiri et al., 2016) and 0.64–1.32% for different brands of orange juice (Ndife et al., 2013). Generally, the juices were rich in carbohydrate and moisture, but low in protein, fibre, fat and ash. The difference between the treatment means was significant at 95% confidence interval (P ≤ 0.05).

The most abundant mineral in the juice samples was potassium followed by calcium and phosphorus. Dosumu et al. (2009) and Ijah et al. (2015) reported similar conclusions on blended juices. The micro minerals Iron (Fe) and Zinc (Zn) were present in trace amounts. Potassium is an essential mineral; it plays a role in transmitting nerve impulses, helps to maintain the body's water and acid balance as an important electrolyte. It deficiency is rare but there is some concern that a high sodium-to-potassium intake may cause high blood pressure. Potassium intake can therefore be increased from consumption of citrus fruits (Whitney and Rolfes, 1999). Generally, deficiencies in micronutrients (minerals) are associated with severe malnutrition conditions and mental impairment (Dosumu et al., 2009).

Phytochemicals are plant chemicals that possess varying degrees of therapeutic activities (Omoregie and Osagie, 2012) and may display their health protective effects in diverse ways. They can act as antioxidants (polyphenols, carotenoids) and protect cells against free radical damage (Omoregie and Osagie, 2012). They also have antibacterial, antimalarial (alkaloids), anti-tumour and anti-viral (tannins) properties (Dua et al., 2013). The total polyphenol content (Table 2) recorded in the juices is much lower than the results of Iqbal et al. (2015) for the methanolic extract of *G. velutinus* leaf and bark (77.7mg and 68.3mg GAE/g) extracts respectively. This is because leaf and bark extracts are generally more concentrated while juice is more dilute. The presence of these phytochemicals in the juices gives them some nutraceutical characteristics (Lawal et al., 2013).

Factors that affect the microbial colonization of juices include redox potential, pH, water activity, nutrients, temperature, antimicrobial agents and relative humidity (Raybaudi-Massilia et al., 2009). In the present study, the juice contained more bacteria than yeast as stated by Rivas et al. (2006). At pH values of 1.5, moulds and yeasts are capable of growth. The pH values ranging from 2.9–3.5, pH 3.0–4, and 3.6–4.5 allow the growth of lactic acid bacteria, acetic acid bacteria and enteric bacteria respectively are higher than those for growth of yeasts (Lawlor et al., 2009). Coliforms are indicators of unhygienic practices, poor quality of source of water used and unsanitary conditions, during or after fruits juice processing. Although rare in fruit juices, their presence has been reported in fruit drinks as a result of the use of contaminated materials, ingredients or environmental factors (Essien et al., 2011). Nevertheless, coliforms have been reported not to be of public health significance in fresh or frozen citrus products (Essien et al., 2011).

Use of antimicrobials for extending the shelf life of juices has been practiced for a long time. The preservative had little effect on the physico-chemical characteristics of the juice. The shelf life of the juice without preservatives lasted three weeks at room temperature except F22 (5% lemon juice). This is a much longer period compared to reports of Yadav et al. (2014) for mulberry juice which lasted only two days without preservative. This could be due to the fact that ginger, lemon and honey have preservative properties and have been used as natural preservatives (Mishra and Behal, 2010). All the juices with preservative lasted four weeks at room temperature except F27 (F27w3p; 10% lemon juice without sugar). For the juices in the refrigerator, those without preservative lasted four weeks except F21 (10% lemon juice with sugar), whereas all those with preservative stayed fresh within the four weeks of storage. F21 spoilage could be as a result of possible contamination from the container in which it was preserved (Essien et al., 2011).

A decrease in pH and vitamin C (Tables 3 and 4) over 1 month of storage at room temperature and in the refrigerator has been reported by Ibrahim (2016) for pawpaw, pineapple and watermelon juices. The decrease in vitamin C (Table 4) observed in the juices with increase storage time could be as a result of the effect of oxidation reactions taking place during storage considering the unstable nature of Vitamin C (Okorie et al., 2009). Despite the decrease in the vitamin C content, the juices stored at refrigeration temperature had higher values because the juice was more stable at the low temperature. Increase in total soluble solid content has been reported to be as a result of solubilisation of fruit constituents during storage while increase in titrable acidity may be due to breakdown of pectin into pectinic acid (Yadav et al., 2014). Increase in total soluble solids indicates proper preservation of the juice whereas a decrease signifies deterioration which may be due to fermentation of carbohydrates (sugars) into carbon dioxide, water and ethyl alcohol (Wisal et al., 2013). Furthermore, breakdown of non-reducing sugars and other polysaccharides increases the reducing sugar content of the juice with increase in storage time. As a result of breakdown due to acid hydrolysis, the non-reducing sugar content of the juice decreased with increase in storage time. A decrease in reducing and non-reducing sugar could also be as a result of microbial absorption of nutrients during fermentation (Wisal et al., 2013). The juices were better preserved in the refrigerator, though a combination of preservatives alongside pasteurization at 100 °C for 20 min has been reported to improve the shelf life stability of mulberry juice for a period of 9 months (Yadav et al., 2014).

## 5 Conclusion

Juice was successfully formulated from a combination of orange, lemon, ginger and honey, and was found to be rich in carbohydrate and moisture, but low in protein, fibre, fat and ash. The most abundant mineral in the juices was potassium followed by calcium and phosphorus. The micro minerals Iron and Zinc were present in trace amounts. Amongst the five best formulated juices, F22 (5% lemon juice) was the most organoleptically accepted. On the other hand, F21 (10% lemon juice + sugar), which had the least overall acceptability amongst the five, was shown to be most nutritive. Combining natural fruit juices could form a better alternative to these artificial products for the betterment of the health of the common man. Orange, lemon, ginger and honey have great potentials in the development of a healthy fruit drink. If well harnessed, this could form a better alternative to the soft drinks flooding our markets which put our health at great risk. This could also be a better way of preserving this products and reducing their spoilage and wastage of resources.

## Declarations

### Author contribution statement

Bernard Tiencheu: Conceived and designed the experiments; Performed the experiments; Analyzed and interpreted the data; Contributed reagents, materials, analysis tools or data; Wrote the paper.

Desdemona Njabi Nji: Performed the experiments; Contributed reagents, materials, analysis tools or data; Wrote the paper.

Aduni Ufuan Achidi: Conceived and designed the experiments; Contributed reagents, materials, analysis tools or data.

Agbor Claudia Egbe: Performed the experiments.

Noel Tenyang: Analyzed and interpreted the data; Contributed reagents, materials, analysis tools or data.

Eurydice Flore Tiepma Ngongang: Analyzed and interpreted the data.

Fabrice Tonfack Djikeng, Bertrand Tatsinkou Fossi: Contributed reagents, materials, analysis tools or data; Wrote the paper.

### Funding statement

This work was supported by Saphi Beverages Scoop, Cameroon.

### Data availability statement

The authors are unable or have chosen not to specify which data has been used.

### Declaration of interests statement

The authors declare no conflict of interest.

### Additional information

No additional information is available for this paper.
